# The meaning of behavioral medicine in the public health field—a review of documents related to medical education in Japan

**DOI:** 10.1186/s13030-016-0058-4

**Published:** 2016-03-02

**Authors:** Shigeru Inoue

**Affiliations:** Department of Preventive Medicine and Public Health, Tokyo Medical University, 6-1-1, Shinjuku, Shinjuku-ku, Tokyo, 160-8402 Japan

**Keywords:** Behavioral science, Medical education, Public health

## Abstract

International standardization of medical education requires Japanese medical schools to restructure their curricula to include “behavioral science.” Two influential documents for Japanese medical education, the “Model Core Curriculum for Medical Education in Japan” and the “Scope of the Japanese National Examination for Medical Doctors” include some key terms regarding behavioral science. However, they are not systematic and the phrase “behavioral science” itself could not be found in these documents. The new global standards for medical education, the “Basic Medical Education WFME Global Standards,” require medical schools to include behavioral science in their curricula. The definition of “behavioral science” in the global standards emphasizes social aspects and determinants of health, which is also a key concept of public health. From the view point of public health, it is hoped that the systematic introduction of behavioral science into Japanese medical education will strengthen the public health mindset of medical doctors, which in turn will support the healthcare system in communities.

## Background

Behavioral science is now drawing a great deal of attention in medical schools in Japan, as a result of the “Basic Medical Education Global Standards (Global Standards)” [1] which was published by the World Federation for Medical Education (WFME). These new global standards require medical schools to include “behavioral science” in their education programs. The Educational Commission for Foreign Medical Graduates(ECFMG)has officially announced that they allow only graduates from medical schools which satisfy these Global Standards to take the examination required for doctors who practice medicine in the US. It is therefore critical for medical schools to satisfy these standards. To that end, the Japanese Society for Medical Education published the Japanese Specifications of the WFME Global Standards [2] and the Japan Accreditation Council for Medical Education (JACME) will accredit Japanese medical schools according to the standards.

The Global Standards include a wide range of items such as mission, educational program, academic staff, resources and governance. (Table 1) Educational program is the second item on the list and includes 8 sub-items. Among those, item 2.3 to item 2.5 are educational categories, more specifically, 2.3 basic biomedical sciences, 2.4 behavioral and social sciences, medical ethics and jurisprudence, and 2.5 clinical sciences and skills. Here, behavioral science is indicated as a sub-item in parallel with basic biomedical sciences and clinical sciences. However, the term “behavioral science” is poorly recognized among academic staff in medical schools. Although there is a sense of bewilderment, there is also a sense of mission in adopting behavioral science into the curriculum. In this regard, and to promote behavioral science education in Japanese medical schools, the Japanese Society of Behavioral Medicine (JSMB) developed a working team and has published the Core Curriculum for Behavioral Medicine in Undergraduate Medical Education.

**Table 1:** The basic medical education WFME global standards [1]

1. MISSION AND OUTCOMES	
2. EDUCATIONAL PROGRAMME	
	2.1 FRAMEWORK OF THE PROGRAMME
	2.2 SCIENTIFIC METHOD
	2.3 BASIC BIOMEDICAL SCIENCES
	2.4 BEHAVIOURAL AND SOCIAL SCIENCES, MEDICAL ETHICS AND JURISPRUDENCE
	2.5 CLINICAL SCIENCES AND SKILLS
	2.6 PROGRAMME STRUCTURE, COMPOSITION AND DURATION
	2.7 PROGRAMME MANAGEMENT
	2.8 LINKAGE WITH MEDICAL PRACTICE AND THE HEALTH SECTOR
3. ASSESSMENT OF STUDENTS	
4. STUDENTS	
5. ACADEMIC STAFF/FACULTY	
6. EDUCATIONAL RESOURCES	
7. PROGRAMME EVALUATION	
8. GOVERNANCE AND ADMINISTRATION	
9. CONTINUOUS RENEWAL	

This article reviews four documents related to Japanese medical education, 1) the Global Standards, 2) the Model Core Curriculum for Medical Education in Japan [3], 3) the Scope of the Japanese National Examination for Medical Doctors [4], and 4) the JSBM Core Curriculum for Behavioral Medicine in Undergraduate Medical Education [5] from the viewpoints of behavioral science, and discusses the impact of the Global Standards and the JSBM Core Curriculum on public health education.

### Review of documents related to medical education from the view point of behavioral science in Japan

#### The basic medical education WFME global standards [1]

“Behavioral science” is presented as a part of social science in these Global Standards, although it was originally a transdisciplinary area bridging basic science such as psychology, social science such as public health and clinical science such as psychosomatic medicine. The standards require medical schools to incorporate behavioral science as a basic standard in education. According to the standards, behavioral and social sciences include biostatistics, community medicine, epidemiology, global health, hygiene, medical anthropology, medical psychology, medical sociology, public health and social medicine. Table 2 shows the details of the descriptions of behavioral science in these standards.

**Table 2:** The basic medical education WFME global standards [1]

2.4 Behavioural and social sciences, medical ethics and jurisprudence	
Basic standards: The medical school must ● In the curriculum identify and incorporate the contributions of the: ■ behavioural sciences. (B 2.4.1) ■ social sciences. (B 2.4.2) ■ medical ethics. (B 2.4.3) ■ medical jurisprudence. (B 2.4.4) Quality development standards: The medical school should ● In the curriculum adjust and modify the contributions of the behavioural and social sciences as well as medical ethics and medical jurisprudence to ■ scientific, technological and clinical developments. (Q 2.4.1) ■ current and anticipated needs of the society and the health care system. (Q 2.4.2) ■ changing demographic and cultural contexts. (Q 2.4.3) *Annotations:* ■ *Behavioural and social sciences* would - depending on local needs, interests and traditions - include biostatistics, community medicine, epidemiology, global health, hygiene, medical anthropology, medical psychology, medical sociology, public health and social medicine. ■ *Medical ethics* deals with moral issues in medical practice such as values, rights and responsibilities related to physician behavior and decision making. ■ *Medical jurisprudence* deals with the laws and other regulations of the health care delivery system, of the profession and medical practice, including the regulations of production and use of pharmaceuticals and medical technologies (devices, instruments, etc.). ■ The *behavioural and social sciences, medical ethics and medical jurisprudence* would provide the knowledge, concepts, methods, skills and attitudes necessary for understanding socio-economic, demographic and cultural determinants of causes, distribution and consequences of health problems as well as knowledge about the national health care system and patients’ rights. This would enable analysis of health needs of the community and society, effective communication, clinical decision making and ethical practices.	

#### The model core curriculum for medical education in Japan [3]

This document, published by the Ministry of Education, Culture, Sports, Science and Technology of Japan, provides the guidelines for Japanese medical education over 6 years. It consists of two parts: the model core curriculum of pre-medical education, which usually accounts for the first 1–2 years of medical school, and the model core curriculum of medical education for the latter 4–5 years. Although the phrase “behavioral science” is not found in this document, many keywords related to behavioral science are found in the core curriculum of “pre-medical education”, which consists of four sections, with Section 4 titled, “Human behavior and psychology”. Keywords include, for example, operant conditioning and respondent conditioning, social learning, motivation, stress, individual differences, communication, and social relationships. In the medical education part of the document, some related contents can be found in the sections on social medicine and psychiatry. Generally, from the view point of behavioral science, psychology is well-represented in this document.

#### The scope of the Japanese national examination for medical doctors [4]

This document is published by the Ministry of Health, Labor and Welfare of Japan. All medical students must pass the National Examination to be qualified as a medical doctor. This document therefore has substantial influence on the development of curricula in medical schools. Although this document does not include the phrases “behavioral science/medicine”, many clinical problems related to behavioral medicine are listed in the section on psychiatry and psychosomatic medicine. In the part on general medicine, large sections on topics such as medical interviews, terminal care, team medicine and lifestyle are separately presented. In recent years, an increasing number of topics from these sections have appeared in questions on the national examination. In general, this document tended to highlight the clinical aspects of behavioral medicine.

#### The JSBM core curriculum for behavioral medicine in undergraduate medical education [5]

In response to the increased importance of behavioral science in Japanese medical schools, the working group of the Japanese Society of Behavioral Medicine (JSBM) developed the Model Core Curriculum for Behavioral Medicine in Undergraduate Medical Education. The working group consists of specialists from the fields of psychology, clinical medicine and social medicine. Based on two-round surveys using the Delphi method, a curriculum of 15 sessions was developed. The specific characteristics of this document are the development of a transdisciplinary process and integration of various fields in the courses. Although the above two Japanese documents, the Model Core Curriculum and the Scope, are a little weak in the boundary areas of behavioral science, this curriculum could fill in the gaps of such areas.

## Discussion

“Behavioral science” is not a well-recognized phrase in the medical community. Inclusion of behavioral science as a sub-heading in the Global Standards informs medical educators about the importance of this transdisciplinary field. As seen in the review of related documents, key words of behavioral science are found scattered among basic, social and clinical sciences. Given the transdisciplinary nature of behavioral science, this is inevitable. However, it should be pointed out that integration of these fields of knowledge has been poorly supported in the current system of medical education. In view of this situation, the Global Standards are influential and are expected to promote this important area. In addition, the newly developed JSBM Core Curriculum integrated these fields of knowledge in a course and are expected to be adopted in Japanese medical schools.

From the view point of public health, emphasis on behavioral science in education is preferable. Winslow defined public health as “the art and science of preventing disease, prolonging life and promoting physical and mental efficiency through organized community efforts”. Generally, public health aims to prevent disease and to improve human health through a wide range of methods, including psychological, social, environmental and political methods, as well as medical interventions. Taking this into account, behavioral science and public health share common approaches for resolving problems. It can rather be argued that behavioral science is a part of public health. In the US, behavioral medicine is one of the 5 major subjects (including epidemiology, biostatistics, environmental health, behavioral science and health policy/care management) in public health schools. Strengthening behavioral science in Japanese medical education is expected to contribute to cultivating public health mindsets, which highlight social and behavioral approaches for resolving health issues, among physicians. It will in turn support community healthcare systems.

The issue of the lack of specialists in medical schools who engage in behavioral science education remains to be resolved. The transdisciplinary nature of the field indicates the necessity of collaboration between specialists in different fields for education. In this regard, the psychosomatic medicine and public health fields need to be the core of collaboration, which would further promote the development of both fields, not only in education but also in practice and research, by creating opportunities for cooperation.

## Conclusion

The major documents related to medical education in Japan do not include the key phrase, “behavioral medicine”. The Global Standards of medical education, which include “behavioral medicine” as a sub-heading, are expected to promote this transdisciplinary field in Japanese medical education and the public health mindset of physicians.

## Abbreviations

ECFMG: the Educational Commission for Foreign Medical Graduates
Global Standards: the Basic Medical Education Global Standards
JACME: the Japan Accreditation Council for Medical Education
JSMB: the Japanese Society of Behavioral Medicine
WFME: the World Federation for Medical Education
